# The Evanescent GacS Signal

**DOI:** 10.3390/microorganisms8111746

**Published:** 2020-11-06

**Authors:** Xavier Latour

**Affiliations:** 1 Laboratory of Microbiology Signals and Microenvironment (LMSM EA 4312), Normandy University (University of Rouen Normandy), 55 rue Saint-Germain, 27000 Evreux, France; xavier.latour@univ-rouen.fr; 2 Research Federation NORVEGE Fed4277, Normandy University, F-76821 Mont-Saint-Aignan, France

**Keywords:** *Pseudomonas*, two-component system, histidine kinase sensor, metabolic switch, communication, quorum-sensing, signaling molecules, lifestyle switch, agroecology, biocontrol

## Abstract

The GacS histidine kinase is the membrane sensor of the major upstream two-component system of the regulatory Gac/Rsm signal transduction pathway. This pathway governs the expression of a wide range of genes in pseudomonads and controls bacterial fitness and motility, tolerance to stress, biofilm formation, and virulence or plant protection. Despite the importance of these roles, the ligands binding to the sensor domain of GacS remain unknown, and their identification is an exciting challenge in this domain. At high population densities, the GacS signal triggers a switch from primary to secondary metabolism and a change in bacterial lifestyle. It has been suggested, based on these observations, that the GacS signal is a marker of the emergence of nutritional stress and competition. Biochemical investigations have yet to characterize the GacS signal fully. However, they portray this cue as a low-molecular weight, relatively simple and moderately apolar metabolite possibly resembling, but nevertheless different, from the aliphatic organic acids acting as quorum-sensing signaling molecules in other Proteobacteria. Significant progress in the development of metabolomic tools and new databases dedicated to *Pseudomonas* metabolism should help to unlock some of the last remaining secrets of GacS induction, making it possible to control the Gac/Rsm pathway.

## 1. Introduction

The ability of an organism to colonize and adapt to an ecological niche is highly dependent on its capacity to perceive and analyze its environment. Two-component systems (TCSs, a detailed list of all abbreviations used in this article is available in Figure S1) are powerful tools, widely encountered in bacteria, that fulfill these functions [1,2]. The structures and subtleties of molecular functioning in the wide diversity of TCS families have recently been reviewed in detail by Jacob-Dubuisson et al. [3] and Zschiedrich et al. [4]. Briefly, TCSs consist of a sensor kinase embedded in the bacterial (inner) membrane and a cytosolic cognate response regulator. After coming into contact with its biotic or abiotic environmental cue, the sensor (known as S) undergoes autophosphorylation, and activates the response regulator (also known as the response activator, or A) by phosphotransfer. The GacS/GacA TCS is highly conserved in many Gram-negative bacteria, but that of the *Pseudomonas* genus has been promoted as an experimental model [5,6,7,8,9]. *Pseudomonas* spp. have considerable potential for adaptation to fluctuating environmental conditions and different hosts, thanks to the plasticity of their large genome [10,11]. The type species of the genus, *P. aeruginosa*, is an opportunistic pathogen of humans and animals that can also cause necrosis in plants, but most *Pseudomonas* species are commonly found associated with plants [12,13]. Some are saprotrophs that may have beneficial effects on plant growth and health, such as *P. chlororaphis and P. fluorescens*, whereas others are redoubtable phytopathogens, such as *P. syringae* and *P. viridiflava* [14,15,16]. The GacS/GacA TCS supports the diversity of behavior in this genus and its hosts, by playing major ecological (e.g., control of fitness, host virulence or protection), physiological (e.g., control of swimming and swarming motilities, stress tolerance, biofilm formation) and metabolic (e.g., control of secondary metabolites and extracellular enzyme production) roles in pseudomonads [6,17,18,19,20,21]. Thus, the GacS/GacA TCS appears to be a master sensor of the microenvironment, operating in both pathogenic and beneficial pseudomonads [18,22,23,24].

The GacS/GacA TCS has been studied since the early 1990s. These studies were initiated essentially by Professor Dieter Haas and his team at the Department of Fundamental Microbiology of the University of Lausanne [25]. This is well illustrated by the long list of references from his laboratory cited in this review. These researchers were the first to assign the acronym “Gac”, for “global activator antibiotic and cyanide synthesis”, to the response regulator GacA, which was described in the “*Pseudomonas fluorescens*” model strain CHA0 [26]. This biocontrol strain has since been reassigned to the more recently described species *Pseudomonas protegens*, which characteristically produces the antimicrobial compounds pyoluteorin and 2,4-diacetylphloroglucinol (DAPG) [27]. During the same epoch, Estelle Hrabak and David Willis of the Department of Plant Pathology of the University of Wisconsin, discovered the existence of the membrane sensor kinase LemA (for “lesion manifestation”) [28,29]. This kinase was identified in the phytopathogen *P. syringae* pv. syringae B278a, and was implicated in bean leaf damage. The connection between LemA (now called GacS) and GacA resulting in the formation of a TCS—which was non-intuitive given that *gacA* is not clustered next to the corresponding sensor kinase gene—was demonstrated in the *P. syringae* pv. syringae model, by the same team [30]. Subsequent studies led to (i) the detection of GacS and GacA analogs in many other model strains and species, and (ii) detailed characterization of the links between the TCS constituents and their downstream regulatory network leading to the activation or inhibition of target genes [6,20]. This downstream regulatory cascade involves the Rsm proteins. RsmA and its paralogs are RNA-binding proteins that modulate translation initiation or alter the stability of target messenger RNAs. For example, RsmA acts as a global posttranscriptional regulator, controlling the expression of more than 500 genes in *P. aeruginosa*, many of which are involved in virulence or biofilm formation and are downregulated [31]. GacA upregulates the expression of several small non-coding RNAs (sRNAs), the number of which depends on the *Pseudomonas* species considered [32]. These sRNAs are posttranslational regulators, as they can sequester the regulatory protein RsmA and other RsmA-like proteins, depending on the strain, thereby blocking their activity [5,32,33,34,35,36,37]. Thus, the activation of GacS suppresses the inhibitory or stimulating effects exerted by Rsm proteins on the expression of their target genes. Based on these findings, it became clear that GacS/GacA TCS functioning needed to be integrated into a more global cellular mechanism, which the authors referred to as the Gac/Rsm signal transduction (regulatory) pathway [5,17,20].

Almost 20 years ago, Stephan Heeb and Dieter Haas [6] concluded one of the first reviews on the GacS/GacA TCS with a statement that: “*the current challenges are to discover the signal molecule(s) specific to the GacS sensor and to identify additional components in this complex regulatory pathway*”. Today, many of the major elements involved in the functioning of the Gac/Rsm regulon have been identified, but the precise nature of the signal activating GacS remains a mystery. This major gap in our knowledge is particularly penalizing, because knowledge of the GacS signal is essential if we are to understand how various strong physiological and ecological adaptations are triggered in numerous bacteria, with a view to controlling these mechanisms, through anti-virulence and smart biocontrol treatments in particular. This review follows three axes for deciphering the structure and function of the GacS signal. First, the architecture and functioning of the GacS sensor kinase, to date the only known pedestal for the GacS signal, were considered. Then, the candidate cues, already suspected to play the role of the GacS signal on the basis of screening for demonstrated or presumed functions in bacterial metabolism and communication, adaptation to the host and lifestyle selection, were evaluated. Finally, the progress made and novel strategies for biochemical characterization of the GacS signal were summarized, leading to establish a plausible profile for the elusive GacS signal.

## 2. GacS Is a Membrane Sensor Centralizing Multiple Environmental Stimuli

### 2.1. Architecture and Functioning of the GacS Sensor

**Figure 1:**
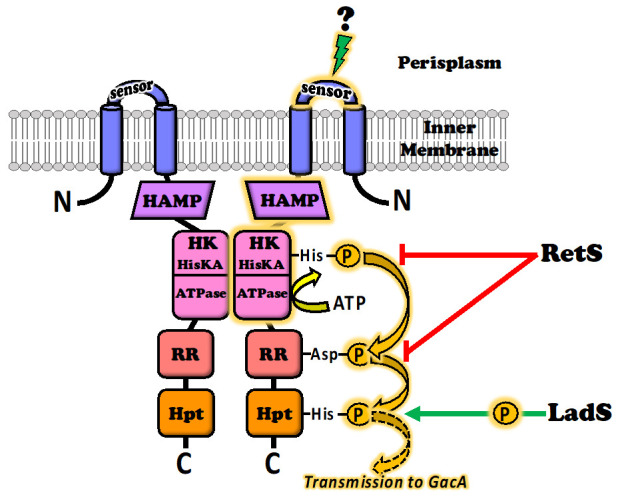
Architecture and functioning of the GacS sensor kinase. GacS is capable of collecting a pool of membrane stimuli: the central environmental source signal (still unknown) is perceived by the periplasmic sensor domain and causes a conformational change that is mechanically transmitted by the HAMP domain to the histidine kinase (HK) domain, leading to autophosphorylation of the key histidyl residue. The phosphorelay involves transmission by the response signal receiver (RR) and histidine phosphotransfer (Hpt) domains, to the cytosolic GacA response regulator. The GacS signal route (marked in amber) is modulated by other environmental cues provided by two other membrane kinase sensors: LadS stimulates GacS activity by shuttling additional phosphate groups into the Hpt domain (green arrow), whereas RetS inhibits GacS activity by siphoning phosphates from the GacS HK, dephosphorylating the RR domain, and/or blocking access to the key histidyl residue of the HK domain (red arrows).

The GacS sensor is a homodimeric protein in which each of the monomers consists of an *N*-terminal transmembrane α-helix, followed by a periplasmic detector domain linked to a second transmembrane α-helix connected to a large cytoplasmic region [6] (Figure 1). The first structural and functional insights into the periplasmic sensor domain were obtained by Zuber et al. [38] in *P. protegens* CHA0. These findings were then clarified by Ali-Ahmad et al. [39] in the *P. aeruginosa* model, in a study based on nuclear magnetic resonance (NMR) spectroscopy and GacS mutants. This study also compared structural sequence alignments of GacS sensor domains with the most closely related kinase sensory domains DcuS from *Escherichia coli* and CitA from *Klebsiella pneumoniae*. Determination of the NMR solution structure of the GacS sensor domain highlighted the presence of a flexible major loop well conserved in the bacterial models studied. This loop has a dynamic conformation in solution, suggesting that ligand binding may induce a conformational change. At the top of the β-sheet facing this loop, three basic amino-acyl residues form an interacting network defining a pocket that could act as a putative ligand-binding site. In *P. aeruginosa*, the functional region consists of the histidyl-97, -124 and -133 residues, which define a positively charged binding-pocket. However, the histidyl-133 residue is not conserved in the DcuS and CitA structural homologs, suggesting that the ligand binding site in GacS is not located in exactly the same place as those of DcuS and CitA [39]. Finally, in silico analyses of more than 200 sequence homologs of the GacS periplasmic sensor domain led to the establishment of a phylogenic tree revealing early divergence between *P. aeruginosa* and *P. fluorescens* within the *Pseudomonas* group [39]. These findings raise questions about the conserved function between the GacS proteins of different species, and about the similarity of the signals recognized by the binding site. These elements will be addressed in Section 4.

The periplasmic domain of GacS plays a crucial role in the sensing and binding of extracellular cues, but the cytoplasmic part of the molecule is also important for the transmission of information to trigger the GacA regulatory response (Figure 1). The GacS cytoplasmic region contains a HAMP phosphatase domain; such domains are present in histidine kinases, adenylate cyclases, methyl-accepting proteins and phosphatases (hence HAMP). The HAMP domain transmits conformational changes from the periplasmic ligand-binding domains to the cytoplasmic part of the protein, more precisely to a histidine kinase (HK) domain. The deletion of this HAMP domain thus results in a strong signal-independent activation of the Gac/Rsm cascade [37]. The HK domain harbors the histidine kinase A, plus a histidine kinase-like ATPase. Its role is to translate the received signal into a phosphorylation event leading to signal transmission. The HK domain can, thus, bind ATP and has histidine kinase activity, leading to self-phosphorylation on a conserved histidyl residue to generate the activated form of the molecule. Interestingly, the HK domain is targeted by ATPase inhibitors from the benzothiazole family [40]. These compounds therefore inhibit the functioning of GacS data transduction and have been considered as a means of controlling TCS activity and associated virulence [40]. Autophosphorylation of the first histidyl residue of the HK domain initiates an aspartyl phosphorelay involving a response signal receiver (RR or REC) domain, followed by a second histidyl residue phosphorelay involving a histidine phosphotransfer (Hpt) domain. Finally, phosphate groups are transferred to a conserved aspartyl residue in the recipient domain of the GacA transcriptional regulator [9,38,41]. The HK and RR domains of GacS also act together in structural interactions of GacS with GacA, and the HAMP domain of GacS is responsible for GacS dimerization. GacS autophosphorylation presumably occurs in trans, within a GacS homodimer [9].

### 2.2. Modulators of GacS Sensor Activity

In pseudomonads, GacS activity is modulated by at least two other inner membrane sensors, called LadS for “loss of adherence sensor” and RetS for “regulator of exopolysaccharide and type III secretion system” [36,41,42,43]. LadS and RetS are also sensor kinases with an *N*-terminal periplasm-facing signal-receiving domain. However, unlike GacS, they are considered to be orphan sensor kinases, because they are not genetically associated with a cognate response regulator. The LadS and RetS sensors do not directly affect the phosphorylation status of the GacS response regulator (i.e., the GacA protein) [43]. They compensate for the absence of a dedicated response regulator by interacting directly with the GacS sensor, to transmit environmental stimuli and integrate their inputs into the Gac/Rsm regulatory pathway. LadS and RetS provide a certain degree of flexibility, because they have counterbalancing effects, with one enhancing GacS signaling, whereas the other attenuates this signaling (Figure 1). LadS stimulates GacS activity by shuttling additional phosphate groups into the Hpt domain of GacS, enhancing the GacS-GacA phosphorelay [44,45]. Conversely, RetS counteracts GacS activity by at least three different mechanisms recently elucidated in the *P. aeruginosa* model. In the first two of these mechanisms, RetS captures phosphate groups from the GacS HK domain, preventing the initial phosphorelay following autophosphorylation from occurring, or dephosphorylates the GacS RR domain. Both these mechanisms prevent the efficient transfer of phosphate groups from GacS to GacA [46]. In the third mechanism, the HK domains of RetS and GacS establish a non-enzymatic tight bond, blocking access to the histidyl residue of the GacS HK domain, thereby preventing its phosphorylation [46,47]. According to the authors, this last mechanism results from the reversible unfolding of a short helical region (helix-cracking) of the RetS HK domain triggered by ligand binding to the periplasmic sensory domain of RetS [47]. Finally, at least on other membrane histidine kinase, PA1611, can also participate in the GacS/LadS/RetS network, by preventing the inhibitory effects of RetS on GacS activation [48,49].

Attempts to identify the signals likely to activate the LadS and RetS membrane sensors have been met with more success than those for the GacS sensor. However, LadS and RetS are characterized by a periplasmic sensor domain different from that of GacS. They carry an *N*-terminal seven-transmembrane domain region with diverse intracellular signaling modules extracellular domain 2 (7TMR-DISMED2) [41,42]. Broder et al. [44] showed that calcium binds to the 7TMR-DISMED2 binding pocket, activating the LadS kinase in *P. aeruginosa*. These authors suggested that calcium is used to sense the intracellular environment of the eukaryote host, to improve the chances of successful long-term host tissue colonization. By contrast, the ligand that binds to the sensor domain of RetS has yet to be fully identified, although the crystal structure of the RetS periplasmic domain revealed the presence of a carbohydrate-binding module [50]. Like LadS, RetS may be responsible for sensing the prevailing conditions in the host. Indeed, RetS can sense unknown cues related to skin cell lysis in *P. aeruginosa* [51]. Moreover, a recent study showed that a component of the mucus coating epithelial cells, mucin glycans, activates RetS via its carbohydrate-binding site in the same model [52]. RetS also appears to be temperature sensitive. Indeed, high temperatures (close to 35 °C) affect antibiotic and cyanide production in *P. protegens* CHA0, possibly via a mechanism involving a change in membrane fluidity, leading to a stronger RetS-GacS interaction [53]. Thus, RetS acts as a temperature-sensitive sensor, inducing a switch in cellular metabolism in conditions of temperature stress for environmental pseudomonad strains, or on contact with warm-blooded host organisms for strains pathogenic to humans and animals.

## 3. Candidate GacS Signal Molecules Identified on the Basis of Their Demonstrated or Speculated Roles

Our ignorance of the identity of the GacS signal is not an isolated case: in many TCSs, the environmental or physiological stimulus triggering the activation of the sensor kinase and, thus, the intracellular regulation cascade, remains unclear [2,4]. The few TCS stimuli identified to date include biotic and abiotic factors, with diverse structures. The affinities of these signals for their sensor kinases range from submicromolar to millimolar values, reflecting the physiological need to trigger a response at a particular signal concentration [2]. The putative stimuli identified encompass simple and complex ions, mineral and organic molecules, including gases, but also solar radiation and physical phenomena acting at the nanoscale, such as the molecular agitation generated by heating. For example, the TodS/TodT TCS detects recalcitrant aromatic compounds in the cell environment, leading to expression of the toluene dioxygenase catabolic pathway [54], whereas DesR/DesK senses decreases in temperature and increases membrane fluidity through Δ5-lipid desaturation [55], and the sensing of blue light by LovK/LovR triggers a mechanism favoring bacterial cell attachment in response to the circadian photocycle [56]. In the face of the profusion of possible sensor kinase-activating candidates, most attempts to identify the GacS signal have been guided by the presumed or demonstrated role of the Gac/Rsm regulon in the model bacterium studied.

### 3.1. The GacS Sensor Kinase as the Trigger of a Metabolic Switch

Pseudomonads are known to have a considerable potential for metabolic adaptation, whether from oligotrophic to rich niches and vice versa, thanks to their highly versatile metabolism [10,11,12,57,58,59,60]. They can assimilate diverse compounds, ranging from highly assimilable sugars, and organic and amino acids to much more recalcitrant molecules, such as hydrocarbons and xenobiotics, depending on the strain considered [12,61,62,63]. The whole metabolism of *Pseudomonas* is centered on the tricarboxylic acid cycle (TCA cycle, also known as the Krebs cycle), which is itself connected to the glyoxylate cycle and the oxygen and nitrogen oxide respiratory pathways [12,64,65,66]. Exceptionally, under anaerobic conditions, most pseudomonads can ferment pyruvate and arginine to generate acetate and lactate plus ornithine, respectively, a strategy that is used for long-term survival and redox balance rather than for growth [64,67]. The primary metabolism pathway of pseudomonads is associated with another based on the production of a wide range of secondary compounds, including cyanide, antibiotics (e.g., DAPG, phenazines, pyoluteorin), cyclolipopeptides, siderophores and phytohormones [68,69,70,71]. The synthesis of secondary metabolites is costly in terms of energy and is therefore affected by basal metabolism and used as an alternative by bacteria in conditions of competitive and nutritional stress [6,72,73]. Secondary metabolites are thought to confer a selective advantage on the organisms producing them, in conditions in which these organisms cannot rely on their full growth potential to compete with other organisms in natural environments [73,74]. By contrast, during culture in synthetic media, these metabolites are not essential to the survival of their producers and are generally produced only during stationary phase, when cell population density is very high, and bacterial growth is restricted [23,68,75].

The Gac/Rsm regulon encodes the components of the master regulatory pathway for secondary metabolite production in pseudomonads [6,20,71]. The switch between primary and secondary metabolism is controlled by the RsmA (for “regulator of secondary metabolism”) and RsmA-like intracellular proteins. In *E. coli*, the RsmA homolog is called CsrA, for “carbon storage regulator”, revealing the connection of this protein to the regulation of basal metabolism [20,32]. A similar connection is observed in *P. fluorescens*, in which energy and carbon metabolism are influenced by Rsm proteins, and an *rsmA/rsmE* double mutant has been show to display slow growth [37]. RsmA and RsmE prevent the translation of transcripts for proteins involved in secondary metabolism, by preventing the ribosome from binding to target mRNAs. This repression is relieved by Rsm sRNAs, which are produced in large amounts under positive regulation by GacS/GacA, which act to neutralize the effects of RsmA and RsmE by titration [6,37]. RsmA can also have a positive effect on the translation of mRNAs encoding proteins involved in the induction of lipase and rhamnolipid synthesis, probably through mRNA stabilization [76]. In return, the functioning of Gac/Rsm seems to be affected by core primary metabolism, particularly as concerns the imbalance between the intracellular concentrations of TCAs [77]. Mutations of the gene encoding pyruvate carboxylase, an enzyme responsible for regenerating the oxaloacetate intensely consumes by the TCA cycle, lead to a downregulation of secondary metabolism in the absence of detectable nutrient limitation. By contrast, a mutation of the gene encoding a fumarase isoenzyme involved in ensuring the supply of malate results in an upregulation of secondary metabolism [77]. The authors showed that these effects were dependent on the GacS sensor kinase, but not on the RetS or LadS sensors, and that they required the production of RsmX, RsmY, and RsmZ sRNAs. Finally, they observed a strong positive correlation between GacA-dependent sRNA production and the intracellular pools of 2-oxoglutarate, succinate and fumarate [77]. This correlation can be explained by the amphibolic role of the TCA cycle, in which certain acids may be catabolized to generate energy, or extracted from the cycle to serve as precursors of secondary metabolites. These observations are also supported by the role assigned to thiamine in this switch [78]. Thiamine is an essential cofactor of TCA cycle enzymes [79]. It is required by pyruvate dehydrogenase, which supplies acetyl-coA to the cycle, and 2-oxoglutarate dehydrogenase, which oxidizes 2-oxoglutarate to generate succinyl-coA. A thiamine auxotrophic mutant derived from *P. protegens* CHA0 was found to have low levels of expression for the RsmX, RsmY and RsmZ sRNAs and for the AprA exoprotease and DAPG antibiotic secondary metabolites, when cultured in the presence of trace amounts of thiamine. The addition of excess exogenous thiamine to the medium complemented all the deficiencies of this mutant. Thus, in the presence of thiamine limitation, functions essential for the primary metabolism of the bacterium can occur, but not several key functions of GacS/GacA-dependent secondary metabolism [78]. These findings led to thiamine and TCA cycle acids being identified and tested as candidate direct or indirect signals activating the GacS sensor. However, none of these molecules seemed to be able to bind to GacS (Table 1).

In *Streptomyces*, another bacterium producing a wide range of antibiotics, the switch between primary and secondary metabolism is underpinned by a transition from glycolytic to oxidative metabolism [88,89]. This probably promotes the production of TCA intermediates required for antibiotic synthesis and increases the efficiency of energy extraction from nutrient substrates required for secondary metabolism. Such a transition would be expected to occur in pseudomonads. Indeed, studies of the mode of glucose assimilation in *Pseudomonas* species have confirmed a preference for organic acids over sugars [60,62,65,66]. Glucose catabolism in pseudomonads is not directed by the conventional glycolysis pathway, but by the Entner-Doudoroff pathway, due, in particular, to a lack of phosphofructokinase, a key enzyme of the glycolytic pathway [60,90]. This results in the prior oxidation of a large proportion of the sugar present by the periplasmic glucose and gluconate dehydrogenases, leading to the production of gluconate and 2-oxogluconate, respectively [91]. Gluconate and 2-oxogluconate are produced under the control of the Gac/Rsm regulon, as is pyrroloquinoline quinone, a cofactor of glucose dehydrogenase [82]. Based on its production close to the periplasmic domain of GacS, gluconate has been considered a potential GacS ligand, as has pyrroloquinoline quinone (Table 1). Unfortunately, other compounds involved in the pathway leading to gluconic acid synthesis, such as 2-oxogluconate, 2-oxo-3-deoxygluconate (KDG) and its phosphorylated form 2-oxo-3-deoxy-6-phosphogluconate (KDPG), have yet to be evaluated. Indeed, KDG, a catabolite resulting from pectin breakdown, is known to act as a signal redirecting energy metabolism towards cell wall compounds in other γ-Proteobacteria, such as *Dickeya* and *Pectobacterium* spp. In *Pseudomonas*, KDGP is the central intermediate metabolite generated by the Entner-Doudoroff pathway. This compound binds the cytosolic HexR regulator, which controls glucose-responsive genes and some functions of central carbon metabolism, releasing the repression of target genes exerted by this protein [60,91].

### 3.2. The GacS Sensor as a Population Density (Quorum) Sensor

Numerous microorganisms use cell-to-cell communication systems based on both the synthesis and perception of signaling molecules to evaluate population density and synchronize their social behavior [72,92]. These quorum-sensing (QS) systems act through the following mechanism: each individual constitutively produces diffusible signals; the environmental concentration of signaling molecules is, therefore, directly linked to the density of the emitting population, but also to the level of cell lockdown favoring signal accumulation. Each member of the population is capable of detecting a critical concentration of signals, corresponding to the cellular quorum. The whole population is therefore, informed of the cellular quorum being reached and of the degree of diffusion from the microenvironment, between bacterial sites within and outside the host, for example. QS molecules generally induce their own production. At high concentration, they stimulate the expression of their synthases. Consequently, the production of QS signaling molecules is often optimal at the end of the exponential growth phase [92]. The best-known QS systems in Gram-negative bacteria produce molecules from the *N*-acyl-L-homoserine lactone (AHL) family [92]. In several *Pseudomonas* species, such as *P. aeruginosa*, *P. chlororaphis* and *P. syringae*, the Gac/Rsm system upregulates the synthesis of AHLs [6,17,23,34,35]. Such upregulation has also been observed in *P. brassicacearum*, at least in the DF41 strain [93,94]. In these bacteria, GacA upregulates the transcription of *luxI/luxR* analog operons, encoding the enzyme synthesizing AHLs and the corresponding AHL response regulators. In addition, *P. aeruginosa* strains use at least two AHL-independent QS systems. The first is based on the secretion and detection of 2-heptyl-3-hydroxy-4(1H)-quinolone, which is usually referred to as the *Pseudomonas* quinone signal (PQS), and its precursor, 2-heptyl-4-quinolone (HHQ) [95,96]. The second uses 2-(2-hydroxyphenyl)-thiazole-4-carbaldehyde QS (IQS) and can integrate environmental stress cues [97]. The PQS and IQS systems are themselves under AHL-based QS control [98], so all of these QS systems are ultimately governed by the GacS/GacA TCS. In other pseudomonads, such as *P. protegens* and most of the strains of the *P. fluorescens-P. putida* continuum, no AHLs have not detected, raising questions about the existence of QS systems in these species [34,99,100,101,102].

At high cell population densities, during the transition from the exponential growth phase to stationary phase, the Gac/Rsm pathway of *Pseudomonas* spp. is activated by uncharacterized molecules present in their own culture supernatants. These molecules induce the phosphorylation of the GacS sensor kinase and, hence, of the cognate GacA response regulator, leading to sRNA transcription [34,35,100,101]. These activators also act as auto-inducers of the Gac/Rsm system via a positive feedback loop. Indeed, very little signal activity is present in the culture supernatants of *P. fluorescens* and *P. aeruginosa gacA* mutants [34,35,84]. More generally, mutants with defects of the Gac/Rsm pathway usually display impaired social behavior [20]. All these traits suggest that the molecules activating GacS are also QS signals acting at the top of the QS signaling hierarchy in AHL-producing pseudomonads, and that the Gac/Rsm system is a QS-like alternative response in the absence of AHLs [38,71]. AHLs have, therefore, been identified as molecules that, if bound to GacS, could inform all pseudomonads of the nearby presence of bacterial populations producing AHLs, in addition to having a possible self-inducing effect in AHL-producing pseudomonads. Intriguingly, the signals extracted from *Pseudomonas* cultures with a high cell density appear to be unrelated to well-known QS signals, such as members of the AHL, PQS or auto-inducer-2 (AI-2) families (Table 1). This is also true for another candidate signal, homoserine lactone, the common core of all AHLs, recently identified as the ligand binding to an AHL-sensor in the *Rhodococcus erythropolis* QS-quencher [103]. Nevertheless, the corollary of the behavior of the GacS signal as an auto-inducer is that a compound with production stimulated by the Gac/Rsm regulon may be the GacS signal.

### 3.3. The GacS Sensor as a Promoter of Host Adaptation

Plants and microbes have evolved together over millions of years and collectively form a holobiont, a complex system involving beneficial, neutral and deleterious interactions between partners [104,105]. In plant-pathogen interactions, the attack on the plant generally begins with a phase of active bacterial multiplication, called primo-invasion, supported by highly assimilable photosynthates released by wounded tissues. A second step involving installation of the disease and its slow progression within the plant then occurs. This phase is less favorable to pathogens, because competition occurs within the dense population of invaders, which is also subjected to the response of the host’s immune defenses [58,62,106,107]. The Gac/Rsm regulatory pathway is thought to be involved in the ecological and metabolic transition of pseudomonad cells arising between these two phases of the disease. *P. syringae* illustrates this assertion well. About 50 pathovars of this pathogen infect almost all economically important crop species, earning *P. syringae* a place among the most pathogenic bacteria on Earth [108]. *P. syringae* is an epiphytic pathogen that infects the internal leaf tissues by swimming through wounds or natural openings, such as the stomata in the leaf surface [15]. It first secretes immunity-suppressing effectors into host cells via its type III secretion system (T3SS), which is crucial for the success of the attack. The GacS/GacA TCS affects the timing of the deployment of the T3SS and the release of effectors in target cells, although the precise timing of these events depends on the strain [8]. This has led to suggestions that some of the plant compounds present in the apoplast might be able to induce the Gac/Rsm regulon. It has been shown that the detection of D-fructose, sucrose and trehalose by *P. syringae*, but also by certain plant growth-promoting rhizobacteria (PGPR), such as *P. fluorescens* C7R12, induces the expression of key *hrpA* and *hrpN* genes involved in T3SS elongation and effector secretion, respectively, in conditions mimicking those of the apoplast [85,87].

In pseudomonads beneficial to the plant, the GacS/GacA TCS also plays a role in bacterial competition in the rhizosphere [22,24]. Thus, the GacS sensor is not specifically dedicated to the transition between virulence stages. The role of seed and root exudates as a nutrient source can account for the spectacular increases in the density and activities of some bacterial populations in the rhizosphere relative to bulk soil [63,104,105]. In the PGPR *P. protegens*, CHA0, exudates released by various monocots and dicots stimulate DAPG antibiotic biosynthesis, which is directly under the control of the GacS/GacA TCS [109]. Other unidentified compounds present in seed exudates can also stimulate the GacS/GacA TCS of *Pseudomonas* sp. DSS73. In this biocontrol strain, exudate from sugar beet seed triggers biosynthesis of the antifungal cyclopeptide amphisin. The synthesis of amphisin in DSS73 is strictly dependent on GacS, and even induction by seed exudates requires a functional *gacS* locus [110]. Finally, it has also been suggested that polyamines and their precursor, *N*-acetyl-glutamate, which are present at high concentrations in root exudates, provide bacteria with information about the nutrient composition of the rhizosphere, as in *P. chlororaphis*, for example, in which the metabolism of these compounds is under the control of the Gac/Rsm network [80] (Table 1).

Secondary metabolites, including phenolic compounds and γ-aminobutyric acid (GABA), are also released in large amounts by the plant during its growth and after wounding, revealing the presence of the plant to the soilborne microflora [111]. Many of these compounds are plant-bacteria signaling molecules [112,113,114]. Single phenolic compounds (i.e., with only one or two aromatic rings), in particular, have been identified as possible candidates for interaction with pseudomonad GacS sensor kinases, because these compounds are already known to interact with GacS analogs encountered in other Proteobacteria phytopathogens. In the soft-rot bacterium *Dickeya dadantii*, *trans*-cinnamic and *o*-coumaric acids, at levels that appear physiologically relevant in plants, have been shown to induce the *hrpA* and *hrpN* T3SS genes, and, thus, to suppress plant defenses. The effect of these phenolic compounds is linked to the Gac/Rsm transduction pathway and requires a functional GacS sensor kinase, suggesting that these phenolic compounds are signals detected by GacS in these bacteria [115]. In *Agrobacterium tumefaciens*, a pathogen that induces tumorous crown galls in a wide range of dicots, the expression of most virulence (*vir*) genes is conditioned by the action of the close GacS/GacA homolog, VirA/VirG, with VirA functioning as a histidine sensor kinase, and VirG as a DNA-binding response regulator. VirA recognizes acetosyringone and its structural analogs. It also responds to specific monosaccharides and low pH, by enhancing *vir* expression [116]. The cytoplasmic linker domain of VirA has been implicated in pH and phenol sensing, but monosaccharides seem to be sensed by the periplasmic domain, which enhances the sensitivity and maximal response to phenolic compounds [117]. In *P. syringae*, some virulence traits are also induced by a mixture of sugar and phenolic compounds: the synthesis of syringomycin and syringopeptin phytotoxins, which are released following plant aggression, is induced by D-fructose and the phenolic sugar arbutin, under the control of the GacS/GacA TCS [86]. According to the speculative model established by the authors, *P. syringae* senses these signaling molecules in the plant apoplast via the GacS sensor kinase, and transmits a relay signal to the GacA response regulator.

### 3.4. The GacS Sensor as a Trigger of Lifestyle Change

Bacteria are only rarely found as single dispersed organisms. They generally live in communities and may colonize the surfaces of minerals and higher organisms, forming biofilms [118]. During this process, they lose motility and attach to surfaces, forming cellular aggregations or microcolonies that are embedded in extracellular polymers providing protection from the surrounding environment. In various *Pseudomonas* species capable of infecting both plant and animal hosts, GacS/GacA functions as a master regulator of multiple virulence traits, including those leading to the crucial switches between motile and sessile lifestyles [8,16,42,119]. *P. syringae* regularly switches back and forth between these lifestyles. *P. syringae* cells live both on mineral and plant surfaces, often in the epilithic and phyllosphere biofilms, respectively [120]. The apoplast is a potential carbohydrate-rich habitat for the bacterium and is targeted by *P. syringae*, but heavily defended by the plant. *P. syringae* therefore has to trigger a succession of lifestyle switches to cause disease, in which it develops motility, toxin and exopolysaccharide production, and the secretion of immunity-suppressing effectors into host cells via its T3SS [7,16,28]. A model has recently been proposed in which the Gac/Rsm operon regulates motility and the T3SS in opposite manners during host infection by *P. syringae* DC3000. In this model, GacS/GacA TCS is activated on the leaf surface, thereby promoting motility and damping down deployment of the T3SS. It is then deactivated during early apoplast colonization, leading to a decrease in motility and an increase in type III secretion to suppress host immunity [8]. The authors of this study also suggested that the GacS/GacA TCS may coordinately regulate additional virulence-associated traits, such as toxin production and biofilm formation, in a similar manner [8]. Thus, the GacS/GacA TCS and ensuing lifestyle switches are triggered by nutrient deficiency, as experienced on the surface of the leaf, and deactivated in conditions of nutrient abundance.

*P. aeruginosa* is a highly prevalent opportunistic pathogen of animals and humans, particularly immunocompromised individuals and cystic fibrosis patients. However, some studies have shown that although this bacterium does not cause damage in field conditions, it can cause black necrosis when used to inoculate aerial parts of the plant, such as the leaves. These symptoms reflect the action of virulence factors altering both human and plant cells, such as phospholipase C, exotoxin A and T3SS, which is known to be produced under the control of the Gac/Rsm regulon [13,14,121]. The switch from a planktonic to a sessile lifestyle is a survival advantage enabling pathogens, such as *P. aeruginosa*, to evade stresses and adverse conditions. The formation of a biofilm may decrease the acute virulence of the pathogen, but it provides *P. aeruginosa* strains with an extremely high capacity to persist against host responses, such as phagocytosis, free radical release by immune cells, and antibiotics used for treatment, in addition to nutrient and oxygen limitation [21]. *P. aeruginosa* switches between the motile and sessile lifestyles, modulating its secretion of virulence effectors by a plethora of TCS, QS networks, sRNAs and transcription factors [21,122,123]. The Gac/Rsm regulatory network acts as the decision-making switch [42,119]. However, the continued functioning and flexibility of Gac/Rsm over time are particularly important during the transition to a sessile lifestyle, because RsmY and RsmZ sRNA levels, and, consequently, RsmA activity, are continually modulated during the process of biofilm development [124]. The RNA-binding protein RsmA downregulates surface attachment, exopolysaccharide production and biofilm maturation, whilst inducing flagellum-based motility, the production of the T3SS, type 4 pili and other virulence factors, such as exotoxins and proteases [21]. RsmA also represses diguanylate cyclases, thereby preventing an increase in intracellular 3′,5′-cyclic diguanylic acid (cyclic di-GMP) levels. Cyclic di-GMP is an almost ubiquitous second messenger present in diverse bacteria. Increases in its levels within cells inhibit motility and trigger biofilm formation. The sensing of environmental cues and stress conditions by the envelope sensors of *P. aeruginosa* triggers the Gac/Rsm cascade. This cascade leads to the generation of the RsmY and RsmZ sRNAs, which counteract the translational repression activity of RsmA, leading to an increase in cyclic di-GMP levels, and, consequently, biofilm formation [31,124].

During pathogenesis *P. aeruginosa* cells in the planktonic state are considered to be equipped for the aggressive primo-invasion of the host required for acute infection. By contrast, the formation of a mucoid biofilm is a hallmark of chronic infections and indicative of disease progression and long-term persistence [21,124]. Calcium has been identified as a first signal by which the Gac/Rsm pathway induces a switch from acute-to-chronic virulence has been identified in *P. aeruginosa* [44] (Section 2.2). This discovery led to the concentrations of ions surrounding the bacteria in the vicinity of the host being considered as important cues, and to the notion of diverse possible roles, including as a potential ligand of the GacS sensor, but without convincing results (Table 1). Another avenue of investigation that has yet to be explored, is the possibility that GacS signal is a typical component of the biofilm initiating its own production or revealing its presence to external bacterial populations, thereby favoring biofilm extension. Such a role has been hypothesized for mannuronic acid and guluronic acid, the monomers of alginate, a major exopolysaccharide component of biofilms. Indeed, alginate production is induced by the Gac/Rsm cascade under conditions of metabolic stress, such as microaerophilia or anaerobiosis, as observed during chronic *P. aeruginosa* infections and in *P. fluorescens* mucoid colonies [123,125]. Moreover, the concentrations of these acids are controlled in the periplasm, the location of GasS signal-binding domain, in which alginates not exported to the microenvironment are degraded. Finally, alginate biosynthesis is costly to the bacterium, due to its requirement of nucleotide sugars and the sequestering of carbon, to the detriment of central metabolism. Biofilm production therefore leads to a switch between cell growth sustained by primary metabolism and the production of exopolysaccharides [125,126].

The Gac/Rsm signal transduction pathway is also involved in the switch between the planktonic and biofilm lifestyles in non-pathogenic *Pseudomonas* spp. [6,20,100,127]. In PGPR strains, this switch enables the bacteria to proliferate on plant roots and, subsequently, to compete effectively with other rhizosphere organisms in a sessile mode of growth [101,128]. Genome-wide transcriptome analysis of *P. fluorescens* SBW25 revealed that a mutation of *gacS* led to changes in the expression of genes involved in biofilm formation, motility, stress responses and survival [19]. The spontaneous mutation of Gac/Rsm system genes in *P. fluorescens* F113 and *P. brassicacearum* NFM421 enhances motility and root colonization, probably due to improvements in the harvesting and use of rhizosphere exudates [101,129]. Similar differences were also observed in laboratory conditions, especially in rich media, in which *gacS* or *gacA* mutants grow well and may even have a temporary advantage over the wild-type [6]. By contrast, Gac mutants are generally poor at biofilm formation. They have a lower capacity for biocontrol and are considered to be less competitive than the wild-type against other microbes, particularly in conditions of nutrient limitation. This defect is imputed to the low levels of secondary metabolites produced by Gac mutants, particularly for HCN and antibiotic biosynthesis [6,74]. Based on these observations, some plant photosynthates and secondary metabolites exuded into the phyllosphere, apoplast or rhizosphere were identified as potential GacS inducers (Table 1). However, the role of each of the molecules studied, particularly in switches to a more efficient lifestyle, is difficult to understand fully, depending on whether the molecules are considered to act as extra- or intracellular signals. This is the case with GABA, a compound produced by numerous bacteria, but also by plant hosts [112]. Kasumi Takeuchi [81] showed that the Gac/Rsm pathway has effects on GABA intracellular accumulation similar to those on secondary metabolism and the production of extracellular enzymes. Curiously, the accumulation of GABA promotes planktonic growth and root colonization, and reduces the formation of biofilms in *P. protegens* CHA0, entirely contrary to expectations. It appears that GABA should not be considered primarily as an extracellular signal monitoring the plant, but rather as an bacterial intracellular signal inhibiting the Gac/Rsm cascade and biofilm formation, with other intracellular effectors such as cyclic di-GMP and guanosine tetra/pentaphosphate ((p)ppGpp), usually having the opposite effect [21,124].

## 4. Strategies for Characterizing the Nature of the GacS Signal

### 4.1. Massive Extraction and Fine Characterization of Self-Produced GacS Signals

The paradigm according to which the GacS signal is used as a means of communication in pseudomonads is based on observations that (i) *Pseudomonas* cells growing at high population densities excrete a compound or compounds activating their own GacS sensor kinase, and (ii) these compounds act as a QS-molecule (see Section 3.2). A mutagenesis strategy was therefore envisaged to target the *gacS* anabolic genes of a *Pseudomonas* GacS signal emitter and to identify them by comparison with existing gene databases. Provided that the genes concerned are relatively small and clustered, as for the AHL and AI-2 biosynthetic genes, it is possible to establish a genomic library for the isolation of genes involved signal synthesis [83]. Conversely, if the genes involved in signal design are too numerous and dispersed throughout the genome, the data may not be suitable for full characterization of the signal molecule. This is the case for the cluster of genes directing the unidentified Vfm-QS signal found in *Dickeya* spp. These genes encode proteins displaying similarities both to enzymes involved in amino-acid activation and enzymes involved in fatty-acid synthesis, but provide no decisive insight into the structure of Vfm signaling molecules [130]. No mutants with specific defects of structural genes for GacS signal synthesis are currently available [20]. Unfortunately, it is not possible to obtain any real additional information on the complexity of GacS signal structure by this approach, because it remains possible that the genes involved in the synthesis of the GacS signal or its precursors are essential for cell metabolism and that all mutations affecting GacS signal synthesis are, thus, lethal.

As an alternative to mutagenesis, analytical biochemistry strategies for identifying GacS signals are based on the extraction and characterization of the molecules trapped in the supernatant of the producing bacteria. Thus, supernatants of *P. protegens* CHA0 cultures grown to late exponential growth phase contain, in addition to the molecules present during the stationary phase, signaling molecules that activate the transcription of the RsmY and RsmZ sRNAs, resulting in the induction of secondary metabolites, such as HCN [33,38,131]. Thanks to this fundamental finding, Christophe Dubuis came very close, during his PhD [83], to identifying a GacS signal produced by the CHA0 strain. He first cultured this model strain at high density in GCM synthetic medium [132]. This medium contains principally glycerol as the basic carbon and energy source, supplemented with casamino acids to satisfy selective nitrogen requirements, buffered with phosphate salts. Casamino acids are a mixture of amino acids derived from fully hydrolyzed casein. The use of GCM medium was based on the hypothesis that none of these amino acids was itself the GacS signal, but that they were likely to support its production. As expected, uninoculated GCM medium did not contain the signal [33]. Moreover, it has since been shown that, when cultured in this medium, *P. aeruginosa* rapidly and completely digests amino acids that can be easily integrated into central metabolic pathways until the mid-exponential growth phase, subsequently digesting others that require a more complex catabolism [58]. Finally, supernatants from the stationary phase mostly lack these amino-acids components [58]. Dubuis [83] subjected a large volume (about 100 L) of culture supernatant to extraction in dichloromethane. The dichloromethane extract was purified by separation on a silica gel column with a linear gradient of methanol. The fractions obtained were then assayed for β-galactosidase activity with *P. protegens* CHA0 (*hcnA’-‘lacZ*) or (*rsmZ-lacZ*) reporter strains, leading to the identification of two different active fractions. The most promising fraction was subjected to chromatography on a Sephadex LH-20 column, with methanol as the solvent. Finally, the constituents of the purest concentrated active fraction were separated by reverse-phase chromatography on a C18 column, by HPLC-MS analysis, or by reverse-phase chromatography followed by GC-MS. The active fraction contained a compound with a molecular mass of *m/z* 278, in which the MS detection of specific fragments suggested the presence of at least two hydroxyl groups [83]. The stability of this GacS signal was evaluated by treating the fraction with proteinase K, boiling for 10 min or exposure to pH 2 and pH 12: these treatments did not affect the activity of extracts [83]. Stability at pH 12 indicates that the signals are not AHLs, because, at this pH, the lactone bond of AHLs is hydrolyzed [133]. If this stability at extreme pH was confirmed, it would exclude from the list of GacS signal candidates the hydroxycarboxylic acids which spontaneously cyclize to form lactones, with a five- or six-membered ring (γ- and δ-lactones). The absence of absorption at 210 and 254 nm, indicates that the extracted signal is unlikely to contain an aromatic ring or conjugated double bonds. A peptide structure is also unlikely, because the signal provided no evidence of amino groups on MS and was not destroyed by proteinase K [83]. Pinolenic acid (CAS number 168-33-54-8), which is found in diverse pine nuts, is an unsaturated aliphatic monocarboxylic acid with 18 carbon atoms and the same molecular mass as the GacS signal extracted from the CHA0 strain. However, this compound has no hydroxyl or ketone groups. According to the work of Dubuis, the GacS signal probably has a structure closely resembling that of this compound. By analogy with its QS functions, it could also have a structure similar to the acyl chain part of AHLs, or to the signaling molecules produced by *Ralstonia* spp. (i.e., the volatile extracellular factor 3-hydroxy-palmitate ester) [134], or by *Xanthomonas* spp. (i.e., the diffusible signal factor *cis*-11-methyl-2-dodecenoate) [135].

Although it was not possible to characterize the active compound present in the CHA0 fraction fully, the GacS induction capacities of the purified fraction were assessed. CHA0 mutants with defects of *gacS* and *gacA* produced signals with a strength about 25- to 30-fold weaker than that of the wild-type [83], confirming that signaling molecules induce their own synthesis in an auto-induction process. Self-produced GacS signals have been found in various *Pseudomonas* species, making it possible to compare signaling both within and between species [83,84]. This work demonstrated the existence of crosstalk responding to the signal extracted from *P. protegens* CHA0. This crosstalk occurs at variable intensities, between strains from the same species, but also between strains from different *Pseudomonas* species (e.g., *P. protegens* CHA0 and *P. aeruginosa* PAO1) or even different genera (*Pseudomonas* and *Vibrio*) [83,84]. These findings suggest a certain ubiquity in GacS signal recognition and, therefore, structure, at least in *Pseudomonas* spp. However, the GacS signal may be due to a family of molecules, such as those of the AHL family, for example, accounting for the different intensities recorded in response to the CHA0 signal.

### 4.2. Metabolomic and Wide Screenings of Potential GacS Signals

The metabolome is the inventory of all the metabolites in a biological system [136]. Metabolites are the end products of cellular regulatory processes, and their levels can be regarded as the ultimate response of biological systems to genetic or environmental changes [136]. The GacS signal therefore corresponds to a typical metabolite excreted into the extracellular metabolome. Metabolomic analysis is usually based on techniques such as NMR spectroscopy, matrix-assisted laser desorption-ionization time of flight (MALDI-TOF) and imaging mass spectrometry (IMS). Liquid-state NMR spectroscopy has been used to characterize the medium in which pseudomonad cells are cultured, to identify differences in excreted metabolites and changes in the amount of compounds present in the medium [137]. It can be used to monitor the metabolic footprint of cultures over the entire growth curve, which is essential when searching for QS signals, for example [137]. However, the sensitivity of classic NMR analysis is relatively limited for the capture of signaling molecules, which are generally present at very low concentrations in crude extracts. NMR spectroscopy on fractions with GacS activity isolated from *P. protegens* CHA0 supernatant was inconclusive, probably because the concentration of signal molecules was too low for significance to be achieved in the analysis [83]. Improvements to NMR spectroscopy, based on techniques such as high-resolution magic-angle spinning (HRMAS), have been used to determine the chemical composition of the cells themselves. Moreover, to promote the separation of metabolites and to increase sensitivity, gas chromatography-mass spectrometry (GC-MS), and liquid chromatography-mass spectrometry (LC-MS) have often been incorporated into NMR studies, for the quantitative profiling of low-molecular weight metabolites in particular [137,138,139]. Finally, MALDI-IMS adds the possibility of analyzing various compounds involved in the interactions of microbial colonies [139,140]. Metabolomic approaches based on this tool have made it possible to visualize metabolic exchanges within and between microbial species, including the production of secondary metabolites by pseudomonads [141,142,143]. Interpretation of the results obtained with metabolomic tools requires the use of powerful spectral databases (e.g., METLIN, MassBank) and appropriate software (e.g., CFM-ID). Metabolomic databases, such as the *Pseudomonas aeruginosa* Metabolome Database (PAMDB), the Golm Metabolite Database (GMD), Metabolights, Global Natural Products Social Molecular Networking (GNPS), and the Kyoto Encyclopedia of Genes and Genomes (KEGG), can be used to explore metabolite structures and pathways in addition to significant genes involved in the biosynthesis of compounds [139,144].

Metabolomic approaches have not yet been used specifically in the search for the GacS signal, but they have been used to study physiological phenomena known to be under the control of the GacS/GacA TCS. The metabolome of wild-type *P. protegens* CHA0, a *gacA*-negative mutant, which has lost Gac/Rsm activities, and a *retS*-negative mutant, which shows strongly enhanced Gac/Rsm-dependent activities, were compared after these strains were grown in a modified GCM medium [145]. This work highlights some intracellular metabolites, the production of which is controlled by the Gac/Rsm cascade. As example, the production of ppGpp and 3-hydroxyaspartic acid are more than six-fold upregulated in *gacA* and *retS* mutants than in the wild-type, respectively [145]. Other studies have made it possible to identify several metabolic pathways that are stimulated or repressed in *P. aeruginosa* (i) during changes in environmental metabolic conditions [58], (ii) under AHL-dependent QS regulation [138,146], (iii) during lifestyles adopted by the bacteria under planktonic or biofilm growth [137], and (iv) during long-term adaptation of the bacteria in the host [62,67,147]. These studies cannot provide crucial information about the nature of the GacS signal, but they have made it possible to confirm the key role of metabolites already identified as influencing and/or being under the influence of the Gac/Rsm regulon. These metabolites are TCA cycle intermediates, amino acids, polyamines and fatty acids, the concentrations of which are affected during the switch studied [58,62,138].

## 5. Conclusions

The Gac/Rsm regulon and its master extracellular sensor, GacS/GacA, are powerful elements controlling the expression of the about 10% of the genes in the genomes of various pseudomonads [17,19,148,149,150]. As a consequence, many studies have attributed diverse roles to the GacS sensor, ranging from bacterial QS to triggering metabolic or lifestyle switches, in response to conditions encountered within or close to the plant, for example. Far from being incompatible, all these functions are tightly interconnected, and can be explained in terms of the nutrient availability. Indeed, the signals activating the GacS/GacA TCS are produced at high cell population densities, triggering the Rsm cascade. This favors the switch from primary to secondary metabolism and from lifestyle, effectively contributing to competition between the GacS signal producer and the native microflora, thereby improving survival or fitness in the ecological niche [73,74,151]. The virulence and biocontrol factors controlled by the Gac/Rsm pathway are highly dependent on population size and can therefore, be regarded as manifestations of social behavior to deal with competition with invaders and the immune responses encountered the host [37,72,139,146,152,153]. Thus, most mutants with defects of the Gac/Rsm pathway have lost part or all of their virulence or have a reduced ability to antagonize fungi and to suppress plant diseases [6,23]. Serendipitously, these physiological and ecological transitions can easily be mimicked under laboratory conditions. During pure culture, a *Pseudomonas* strain reaching the late stages of the exponential growth phase produces a critical quantity of GacS signal. This suggests that the nature of the compounds activating GacS is linked to the appearance of nutritional stresses during restricted growth at high cell population density. This observation is supported by the positive regulation of the Gac/Rsm system by ppGpp, an intracellular signal characteristic of nutrient limitation and stress [145]. Moreover, (p)ppGpp is also known to be integrated into QS signaling networks, which ensure that costly virulence factors are produced at the appropriate cell density and under conditions of nutrient limitation [21,154]. The hypothesis that the GacS signal monitors metabolic stress might also account for the profusion of potential signal sources proposed in previous studies. During the hostile conditions imposed by competition for nutrients, GacS signals may be produced not only by pseudomonad populations, but also by the indigenous microbial community and/or the eukaryote host. Compounds with a profile of this type would be diffusible catabolites that may or may not be converted into signals by pseudomonads, with concentrations increasing in the microenvironment in a density-dependent manner. Such a mechanism of metabolic transition has recently been described in *P. protegens* strain Pf5, in which an intermediate catabolite produced during DAPG synthesis is converted into cell-cell communication signals by halogenation, in turn activating the independent pyoluteorin anabolic pathway [153].

Structural analysis has shown that the GacS sensor kinase acts as an antenna, receiving an extracellular signal, the impact of which is influenced by subservient membrane modules indicating the presence of the host. The periplasmic GacS ligand probably corresponds to a molecule or family of molecules with a low molecular weight, facilitating passage across the outer membrane of the bacterial wall and binding in the narrow pocket identified in the periplasmic domain of GacS. This ligand may carry one or more negative charges capable of interacting with the positive charges encountered in the ligand-binding site, thus favoring such interactions. For example, TCA cycle components, such as citrate, succinate and fumarate, have been shown to act as ligands of the narrow binding pocket located on the periplasmic sensor domains of their sensor kinases, CitA in *K. pneumoniae*, DcuS in *E. coli*, and DctB in *Vibrio cholerae*, respectively [155,156]. A screening strategy for competitive inhibitors targeting the periplasmic binding site of GacS may facilitate advances in research, by making it possible to deduce the structure of the GacS signal from that of its inhibitors.

Attempts to purify the GacS signal produced by *P. protegens* CHA0 showed that it could not be a previously characterized QS molecule, or a small protein or peptide, and that it did not appear to contain aromatic rings [83]. This last finding rules out plant phenolic candidates, at least in this strain. The same study revealed that the GacS signal was a relatively simple and small molecule, apolar but moderately volatile, as revealed by GC-MS purification. It probably consists of an aliphatic chain with oxygen substituents [83]. Taking into account all these considerations, aliphatic organic acids are serious candidates for the chemical class of the GacS signal. These compounds are often hydrophobic and, therefore, cell diffusible, depending on the length and degree of unsaturation of their chain. Like other signaling molecules (e.g., AHLs), they can indirectly monitor extra- and intracellular pH changes according to their level of protonation, with pH an abiotic factor known to vary with cell metabolism. Finally, the number and locations of hydrogen substitutions by hydroxyl or ketone groups harbored by the aliphatic chain of the acid would provide the typical features of the molecule necessary to characterize the role and specificity of the signal, as for the *N*-acyl chain of AHLs.

The increasing development of dynamic and versatile metabolite identification tools may make it possible to identify the family and structure of the GacS signal fully in the near future. It will then be necessary to determine the effective physiological concentration of the GacS signal according to environmental conditions, and its impact on the host and other microorganisms. Finally, these advances should make it possible to establish GacS signal-quenching strategies, similar to those used to control the virulent AHL-based QS communication of certain Gram-negative pathogens [103,157,158,159]. Such clinical and agroecological approaches, recently referred to as “bacterial informational wars” [160,161], might raise a new hope for reducing the Gac/Rsm pathway-based virulence of pathogens. Conversely, the addition of GacS signals or biostimulating molecules (e.g., cheap GacS agonists) could trigger and increase the Gac/Rsm-dependent biocontrol exerted by some PGPRs.

## Figures and Tables

**Table 1:** GacS signal candidates previously studied in pseudomonads.

Candidates	Production under Gac/Rsm Control	GacS Reporter Induction	GacS Periplasmic Domain Binding	Reference
**Metabolites expected to induce a metabolic switch**				
TCA cycle and related compounds:				
L/D Lactate	^1^	^1^	**-** ^1^	[39]
Pyruvate			**-**	[39]
Acetate			**-**	[39]
Citrate			**-**	[39]
2-Oxoglutarate	**+**		**-**	[39,77]
Succinate	**+**		**-**	[39,77]
Malate			**-**	[39]
Fumarate	**+**		**-**	[39,77]
Dicarboxylic acids and TCA antagonists:				
Formate			**-**	[39]
Malonate			**-**	[39]
Glutaraldehyde			**-**	[39]
Tartrate			**-**	[39]
Amino acids and polyamines:				
Glutamate			**-**	[39]
*N*-Acyl-glutamate	**+**		**-**	[39,80]
Glutamine			**-**	[39]
Leucine			**-**	[39]
Arginine	**+**		**-**	[39,80]
Putrescine	**+**		**-**	[39,80]
Spermidine	**+**		**-**	[39,80]
γ-Amino-butyric acid (GABA)	**+**			[81]
Antioxidants, vitamins and coenzymes:				
Glutathion (reduced form)			**-**	[39]
Glutathion (oxidized form)			**-**	[39]
Guanosine diphosphate (GDP)			**-**	[39]
Pyrroloquinoline quinone	**+**		**-**	[39,82]
Thiamine	**+**		**-**	[39,78]
**Quorum-sensing signaling molecules**				
Auto-inducers and related compounds:				
*N*-Acyl-L-homoserine lactones (AHLs, AI-1)	**+**	**-**		[33]
Homoserine lactone (AHL core)			**-**	[39]
*O*-Succinyl-L-homoserine			**-**	[39]
Furanosyl diester (AI-2)	**-**			[33]
γ-Butyrolactones:				
2-Ethyl-4-hydroxy-5-methyl-3(2H) furanone		**-**		[83]
3-Decyldehydro-2(3H) furanone		**-**		[83]
Dihydro-5-propyl-2(3H)-furanone		**-**		[83]
4,5-Dihydro-5-pentyl-2(3H)-furanone		**-**		[83]
4,5-Dihydro-5-heptyl-2(3H)-furanone		**-**		[83]
Dihydro-5-octyl-2(3H)-furanone		**-**		[83]
*Pseudomonas* and *Burkholderia* spp. signals:				
2-Heptyl-3,4-dihydroxyquinoline (PQS)	**-**	**-**		[83,84]
*Ralstonia* spp. signal:				
3-Hydroxy-palmitate methyl ester	**-**			[83]
*Xanthomonas* spp. signal and related acids:				
*cis*-11-methyl-dodecenoate	**-**			[84]
*cis*-2-dodecenoate		**-**		[83]
*trans*-2-dodecenoate		**-**		[83]
*cis*-2-undecenoate		**-**		[83]
Undecanoate		**-**		[83]
**Dications and enzyme cofactors**				
Ca^2+^			**-**	[39]
Mg^2+^			**-**	[39]
Mn^2+^			**-**	[39]
Ni^2+^			**-**	[39]
Zn^2+^			**-**	[39]
**Plant host compounds (cues)**				
Sugar photosynthates:				
Glucose (reduced form)	**-**		**-**	[39]
Gluconate (oxidized form)	**+**		**-**	[39,82]
2-oxogluconate				[82]
Fructose	**+**			[85,86]
Sucrose	**+**			[87]
Trehalose	**+**			[87]
Phenolic compounds:				
Arbutin	**+**			[86]

^1^**-**, no effect; **+**, positive effect; blank, not determined.

## Supplementary Materials

The following are available online at https://www.mdpi.com/2076-2607/8/11/1746/s1, Figure S1: List of abbreviations used throughout this review.
