# Supplementary material for: The Evanescent GacS Signal
Source: Microorganisms. 2020 Nov 6;8(11):1746. doi: 10.3390/microorganisms8111746 (PMC7695008; doi:10.3390/microorganisms8111746)
Supplement: Supplementary file 1 [file microorganisms-08-01746-s001.pdf]

microorganisms

MDPI

Review

The evanescent GacS signal

Xavier Latour [1,2,*]

[1] Laboratory of Microbiology Signals and Microenvironment (LMSM EA 4312) - University of Rouen Normandy - 55 rue Saint-Germain, 27000 Evreux, France
[2] Research Federations NORVEGE Fed4277 & NORSEVE - Normandy University – France & Canada
* Correspondence: xavier.latour@univ-rouen.fr (X.L.)

Supplementary Material

Figure S1. List of abbreviations used throughout this review

AHL	: *N*-Acyl-L-Homoserine Lactone
AI-2	: Auto-Inducer-2
CFM-ID	: Competitive Fragmentation Modeling for metabolite Identification
CitA	: Citrate-malate A (sensor)
CsrA	: Carbon storage regulator A
Cyclic di-GMP	: 3′,5′-Cyclic diguanylic acid
DAPG	: 2,4-Diacetylphloroglucinol
DcuS	: C4-Dicarboxylate uptake S (sensor)
DesK/DesR	: Δ5 acyl lipid Desaturase (histidine kinase sensor, K; response regulator, R)
DctB	: C4-Dicarboxylate transport B (sensor)
GABA	: Gamma-Amino-Butyric Acid
Gac	: Global activator antibiotic and cyanide synthesis
GCM	: Glycerol Casamino acids Medium
GC-MS	: Gas Chromatography-Mass Spectrometry
GMD	: Golm Metabolite Database
GNPS	: Global Natural Products Social molecular networking
HAMP	: Histidine kinases, Adenylate cyclases, Methyl-accepting proteins and Phosphatases
HexR	: Hexoses R (regulator)
HHQ	: 2-Heptyl-4-Quinolone
HK	: Histidine Kinase
HPLC-MS	: High-Performance Liquid Chromatography-Mass Spectrometry
Hpt	: Histidine phosphotransfer
HRMAS	: High-Resolution Magic-Angle Spinning
Hrp	: Hypersensitive reaction and pathogenicity
IQS	: Integrated Quorum-sensing System
IMS	: Imaging Mass Spectrometry
KDG	: 2-Keto-3-Deoxy-Gluconate
KDPG	: 2-Keto-3-Deoxy-6-Phospho-Gluconate

microorganisms

Figure S1. *Cont.*

KEGG	: Kyoto Encyclopedia of Genes and Genomes
LadS	: Loss of adherence S (sensor)
LC-MS	: Liquid Chromatography-Mass Spectrometry
LemA	: Lesion manifestation A (sensor)
LovK/LovR	: Light, oxygen and voltage (histidine kinase sensor, K; response regulator, R)
LuxI/LuxR	: Luminescence (inducer synthase, I; response regulator, R)
MALDI-TOF	: Matrix-Assisted Laser Desorption-Ionization-Time Of Flight
METLIN	: Metabolome Informatics
MS	: Mass Spectrometry
NMR	: Nuclear Magnetic Resonance
PAMDB	: Pseudomonas aeruginosa Metabolome Database
PGPR	: Plant Growth-Promoting Rhizobacteria
(p)ppGpp	: Guanosine tetra- and pentaphosphate
PQS	: Pseudomonas Quinone Signal
QS	: Quorum-Sensing
RetS	: Regulator of exopolysaccharide and type III secretion system S (sensor)
RR	: Response signal Receiver
Rsm	: Regulator of secondary metabolism
sRNAs	: small non-coding RNAs
7TMR-DISMED2	: seven-Transmembrane domain Region with Diverse Intracellular Signaling Modules Extracellular Domain 2
TCA	: Tricarboxylic Acid
TCS	: Two-Component System
TodS/TodT	: Toluene dioxygenase (sensor, S; response regulator, T)
T3SS	: Type III Secretion System
VirA/VirG	: Virulence (sensor, A; response regulator, G)
Vfm	: Virulence factor modulating
